# Swelling, Protein Adsorption, and Biocompatibility of Pectin–Chitosan Hydrogels

**DOI:** 10.3390/gels10070472

**Published:** 2024-07-17

**Authors:** Sergey Popov, Nikita Paderin, Elizaveta Chistiakova, Alisa Sokolova, Ilya V. Konyshev, Vladislav S. Belozerov, Andrey A. Byvalov

**Affiliations:** Institute of Physiology of Federal Research Centre “Komi Science Centre of the Urals Branch of the Russian Academy of Sciences”, 50 Pervomaiskaya Str., 167982 Syktyvkar, Russia; paderin_nm@mail.ru (N.P.); kvashninova.e@yandex.ru (E.C.); alisasokolova.ru@yandex.ru (A.S.); konyshevil@yandex.ru (I.V.K.); belozerovvs@mail.ru (V.S.B.); byvalov@nextmail.ru (A.A.B.)

**Keywords:** pectin, chitosan, hydrogel, swelling, biocompatibility, protein adsorption, leukocyte adhesion, optical tweezers, force of interaction

## Abstract

The study aims to determine how chitosan impacts pectin hydrogel’s ability to attach peritoneal leukocytes, activate complement, induce hemolysis, and adsorb blood proteins. The hydrogels PEC-Chi0, PEC-Chi25, PEC-Chi50, and PEC-Chi75 were prepared by placing a mixture solution of 4% pectin and 4% chitosan in a ratio of 4:0, 3:1, 2:2, and 1:3 in a solution of 1.0 M CaCl_2_. Chitosan was found to modify the mechanical properties of pectin–calcium hydrogels, such as hardness and cohesiveness-to-adhesiveness ratio. Chitosan in the pectin–calcium hydrogel caused pH-sensitive swelling in Hanks’ solution. The PEC-Chi75 hydrogel was shown to adsorb serum proteins at pH 7.4 to a greater extent than other hydrogels. PEC-Chi75’s strong adsorption capacity was related to lower peritoneal leukocyte adherence to its surface when compared to other hydrogels, showing improved biocompatibility. Using the optical tweezers approach, it was shown that the force of interaction between pectin–chitosan hydrogels and plasma proteins increased from 10 to 24 pN with increasing chitosan content from 0 to 75%. Thus, the properties of pectin–calcium hydrogel, which determine interactions with body tissues after implantation, are improved by the addition of chitosan, making pectin–chitosan hydrogel a promising candidate for smart biomaterial development.

## 1. Introduction

A contemporary technique involves employing hydrogels to manufacture scaffold materials for tissue engineering and wound healing. This is because hydrogel structures and functions are similar to those of the extracellular matrix [1,2]. Natural polysaccharides for hydrogel manufacture include advantages such as bioactivity, low toxicity, biocompatibility, and structural and functional resemblance to extracellular matrix glycans [3,4]. It is widely acknowledged, however, that the complexity and dynamism of the microenvironment of an organ or tissue in vivo appear to be beyond the scope of any hydrogel made of a single polymer. It is therefore advisable to develop hydrogels in which two or more biopolymers interact synergistically enhancing the performance of single-polymer hydrogels [5,6]. Among hybrid systems composed of natural polysaccharides, pectin–chitosan hydrogels have recently been proposed for tissue engineering and drug delivery applications. The interaction between the two polysaccharides leads to the formation of a physical hydrogel with the development of an interpolymer complex network [5].

Pectin, a gelling heteropolysaccharide extracted from higher plants, is widely employed in a variety of scientific fields [7,8]. Pectins are polyanionic polysaccharides made up of 1,4-linked α-D-galacturonic acid (GalA) residues that can be methyl-esterified to varying degrees. Pectins with a low degree of methyl esterification form a gel in the presence of calcium cations [9]. The carboxyl group (-COOH) of pectin’s GalA can dissociate into the -COO^−^ group, generating electrostatic interactions with oppositely charged molecules. Chitosan is a natural polymer composed of β (1–4)-linked D-glucosamine and N-acetyl-D-glucosamine residues. It is comparable to glycosaminoglycans found in the extracellular matrix [10]. Under specific pH conditions, the amino groups of chitosan can convert to positive groups (-NH_3_^+^) in the aqueous environment, resulting in electrostatic contact with pectin’s negative carboxyl groups. In recent decades, pectin–chitosan composite materials have been widely studied for their ease of biomedical use [11]. In our laboratory, we have succeeded in preparing pectin–chitosan gel and found that the inclusion of chitosan improved the tissue adhesiveness of pectin gel cross-linked with calcium ions [12].

Both pectin and chitosan are biocompatible [13,14]. However, the high bioadhesiveness of chitosan, including against proteins and cells, makes it vital to research the biocompatibility of chitosan-containing materials. Serum protein adsorption, for example, is among the initial reactions that take place when implanted biomaterials interact with blood [15]. Non-specific protein adsorption is associated with the formation of a foreign body response, which prevents tissue engineering scaffolds from engrafting successfully [16]. On the other hand, interactions between biomaterial surfaces and proteins may promote the integration of the device into the biological environment. For example, improved protein adsorption on the surface of bone substitute materials has been reported to enhance osteoblast attachment and proliferation. Therefore, protein adsorption needs to be assessed both from the point of view of biocompatibility and to optimize the clinical application of the biomaterial. Many studies have shown the ability of chitosan biomaterials to adsorb blood proteins [17,18,19,20,21]. However, the adsorption of blood proteins and the biocompatibility of pectin–chitosan hydrogels have not been studied previously.

This study aims to determine how chitosan impacts pectin hydrogel’s ability to attach peritoneal leukocytes, activate complement, induce hemolysis, and adsorb blood proteins. For the first time, the interaction between pectin and pectin–chitosan hydrogels and plasma proteins was assessed using the optical tweezers method. The phenomenon of proteins interacting with material surfaces is still unknown, although several approaches to monitor the adsorption process have been developed [22,23]. Notwithstanding the significance of these methods, a deeper comprehension of protein adsorption on biomaterials may require measuring the force of protein–surface interactions in addition to measuring the amount of protein adsorbed.

## 2. Results and Discussion

### 2.1. Effect of Chitosan on Mechanical Properties and Swelling Behavior of Pectin–Calcium Hydrogel

The hydrogels, designated PEC-Chi0, PEC-Chi25, PEC-Chi50, and PEC-Chi75, were prepared by mixing 4% solutions of pectin and chitosan in a ratio of 4:0, 3:1, 2:2, and 1:3, respectively. The hydrogels had a light, opaque appearance.

**Figure 1:**
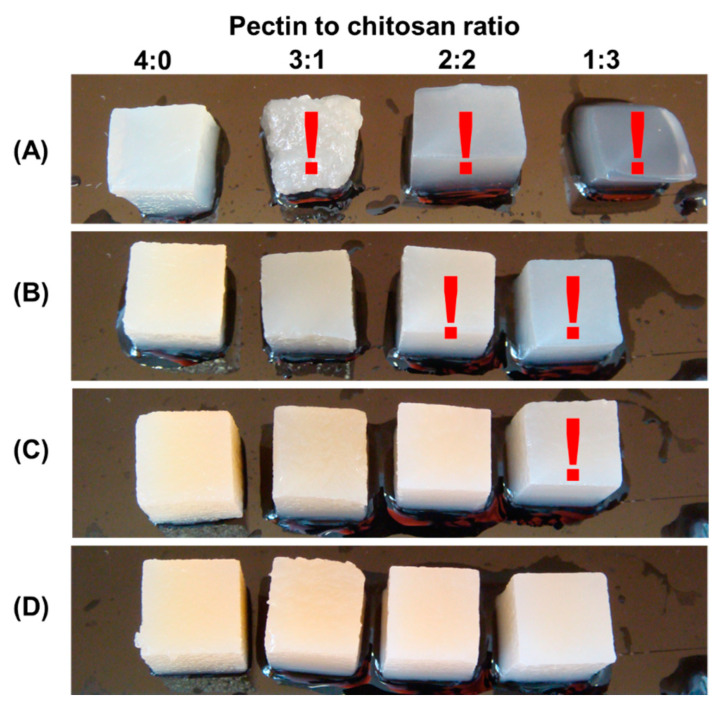
The appearance of hydrogel samples obtained from a mixture of 1% pectin solution and 1% chitosan solution (**A**), a mixture of 2% pectin solution and 2% chitosan solution (**B**), 3% pectin solution and 3% chitosan solution (**C**), and 4% pectin solution and 4% chitosan solution (**D**) at a pectin–chitosan ratio of 4:0, 3:1, 2:2, and 1:3. Samples that are too soft and difficult to work with by hand are marked with a red exclamation mark.

In a preliminary experiment, it was discovered that several composite gels created from 1, 2, and 3% polysaccharide solutions were too soft and difficult to work with by hand (Figure 1). In particular, when 1% polysaccharides were mixed with pectin–chitosan (*v*/*v*) ratios of 3:1 and 1:3, the hydrogels lost their shape and deformed under their own weight. Hydrogels prepared from 2% polysaccharides in a ratio of 2:2 and 1:3 (*v*/*v*) were too soft to allow manual manipulation. The same was true for the hydrogel prepared with a mixture of 3% pectin and 3% chitosan in a 3:1 (*v*/*v*) ratio.

The use of varying pectin–chitosan ratios allowed us to control the number of chitosan chains with free amide groups accessible for interaction with biological targets. According to Formulas (1) and (2), the amount of COO- and NH_3_^+^ groups in the PEC-Chi25 hydrogel was calculated to be equal to 93 and 50 mM, respectively. In this case, all amide groups of chitosan appeared to be occupied by the formation of polyelectrolyte bonds with carboxyl groups of pectin at a pectin–chitosan ratio of 3:1. The PEC-Chi50 hydrogel had 62 and 100 mM COO- and NH_3_^+^ groups, respectively, indicating that there were 38 mM chitosan amine groups available for potential interactions. The PEC-Chi75 hydrogel had 31 and 150 mM COO- and NH_3_^+^ groups, respectively, indicating that there were 119 mM chitosan amine groups available for potential interactions with biological targets.

ν(-COO^−^) = (m(p)/M(p)) × (1 − DM), (1)

ν(-NH_3_^+^) = (m(ch)/M(ch)) × (DD), (2)

Here, ν(-COO-) is the amount of substance with free carboxyl groups; m(p) is the weight of dissolved pectin; M(p) is the molecular weight of galacturonic acid; DM is the degree of methyl esterification; ν(-NH_3_^+^) is the amount of substance with free amine groups; m(ch) is the weight of dissolved chitosan; M(ch) is the molecular weight of D-glucosamine; and DD is the degree of deacetylation.

**Figure 2:**
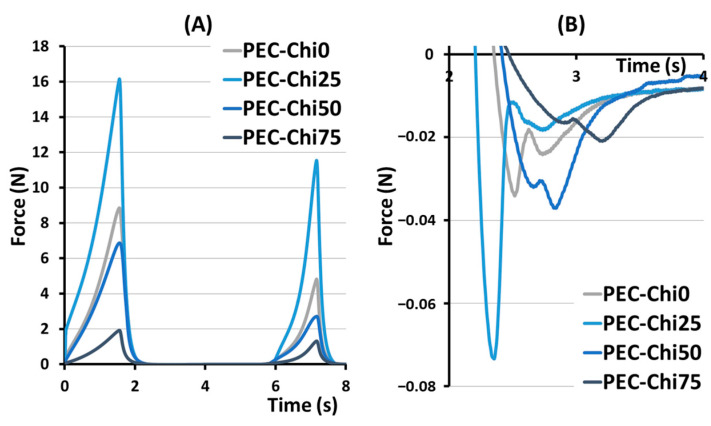
Representative force–distance curves for PEC-Chi hydrogels in a double compression test (**A**). The area under the force–distance curve, which corresponds to the adhesiveness of PEC-Chi hydrogels (**B**).

The hardness, springiness, cohesiveness, and adhesiveness of the hydrogels prepared were evaluated in a double compression test (Figure 2). The effect of chitosan on hardness, that is, the ability of the gel’s structure to withstand compression, depended on its content in the hydrogel (Table 1). The hardness of the PEC-Chi25 hydrogel with a minimal chitosan content was 82% higher than that of the PEC-Chi0 pectin hydrogel. A further increase in the chitosan content in the hydrogel led to a decrease in hardness of 22 and 73% compared to pectin gel for PEC-Chi50 and PEC-Chi75, respectively. Hydrogels exhibited near springiness, which is the capacity to spring again after they were deformed. The PEC-Chi75 hydrogel exhibited 16% more springiness than the PEC-Chi0 hydrogel. The cohesiveness of the pectin hydrogel, which reflects the intermolecular attraction by which the polymer chains are held together, did not change with the addition of chitosan. The adhesiveness of the PEC-Chi25 hydrogel, which reflects its aptitude for interfacial adhesion, was nearly double that of the PEC-Chi0 pectin hydrogel. Increasing the chitosan concentration of the hydrogel to 75% reduced adhesiveness by 42% compared to PEC-Chi0.

The stability of hydrogels was studied during incubation in distilled water at 4 °C for 3 days (Table 2). After one day of incubation, the weight of PEC-Chi0, PEC-Chi25, PEC-Chi50, and PEC-Chi75 hydrogels decreased by 2.8, 6.3, 5.8, and 6.9%, respectively, compared to the initial weight taken as 100%. After that, the weight did not change. The hydrogel showed stability because it did not collapse within 3 days.

It was previously shown that the formation of pectin–chitosan hydrogel was most likely caused by the cross-linking of pectin chains with calcium ions, as well as the formation of a polyelectrolyte complex between pectin and chitosan chains [12]. In the presence of calcium ions, two neighboring chains of low-methyl-esterified pectin bond together to create the “egg box” structure, which offers two ionic connections between free carboxylic acid groups. An ionic bond arises between the pectin carboxylic groups and the chitosan amino groups in pectin–chitosan hydrogels. The pectin–chitosan ratio determines the number of cross-links between the carboxylic groups of two pectins and the cross-links of pectic carboxylic and chitosan amide groups. The hydrogels obtained could be classified into three types of gel networks. The network of PEC-Chi0 was generated solely by the calcium egg box formation, which connected two pectin chains. The network in PEC-Chi25 gel appeared to be formed by the calcium ion cross-linking of pectins as well as the cross-linking of pectin and chitosan molecules via COO- and NH_3_^+^ bonds. In PEC-Chi50 and PEC-Chi75 hydrogels, the gel network was primarily generated by crosslinking pectin and chitosan molecules via COO- and NH_3_^+^ linkages.

The hardness of the PEC-Chi25 hydrogel with a minimal chitosan content was 82% higher than that of the PEC-Chi0 pectin hydrogel. The hardness of PEC-Chi25 hydrogel increased because the ionic bonds between the amino groups of chitosan and the carboxylic groups of pectin were formed in addition to the cross-linking of pectin molecules by calcium ions [12]. A further increase in the chitosan content in the hydrogel led to a decrease in hardness of 22% to pectin gel for PEC-Chi50. This was explained by the fact that the overall number of cross-links in PEC-Chi50 dropped as the chitosan concentration increased since the number of positively charged amide groups exceeded the number of carboxyl groups as the pectin content declined. The hardness of PEC-Chi75 hydrogel was 75% lower than that of PEC-Chi0. The high chitosan concentration of the PEC-Chi75 hydrogels appeared to cause the repulsion of unbound positively charged amide groups, hence weakening the gel network.

The double compression test was used to determine cohesiveness and adhesiveness, which are important textural parameters for predicting the behavior of hydrogel materials. A compromise between cohesive and adhesive forces is believed to be important for the effectiveness of adhesive biomaterials [24]. Changes in the gel network caused by the inclusion of chitosan modified the relationship between the cohesiveness and adhesiveness of the hydrogel prepared. The PEC-Chi0 hydrogel had a cohesiveness-to-adhesiveness ratio of nearly 1, but the PEC-Chi25 and PEC-Chi50 hydrogels had ratios of 0.6 and 0.7. Importantly, cohesive failure can occur when the attractive forces between the hydrogel and the target surface exceed cohesive interactions. On the contrary, cohesive strength exceeded adhesiveness by 2.2 times in the PEC-Chi75 hydrogel. However, cohesive interactions are essential only to a certain amount because too much cohesion can result in a hardened material that is prone to interfacial failure [24].

**Figure 3:**
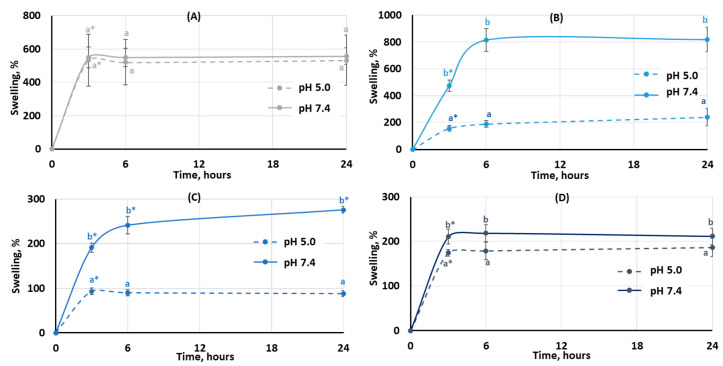
The swelling behavior of the PEC-Chi0 (**A**), PEC-Chi25 (**B**), PEC-Chi50 (**C**), and PEC-Chi75 (**D**) hydrogels during 24 h incubation in Hanks’ solution with pH 5.0 and 7.4 at a temperature of 37 °C. The values (m ± SD, n = 6) are significantly different between pH conditions (*p* < 0.05), as indicated with different lowercase letters. *—*p* < 0.05 compared to the previous time point.

The degree of swelling is important for protein adsorption and subsequent events that occur after biomaterial implantation. The swelling behavior of the hydrogels was studied by incubating dried samples in Hanks’ solution with pH 5.0 and 7.4 (Figure 3). It was found that the degree of swelling of pectin hydrogel not containing chitosan (PEC-Chi0) did not depend on pH. The weight of PEC-Chi0 increased 5–6 times after 3 h of incubation and then remained at this level for 24 h (Figure 3A). The swelling behavior of hydrogels containing chitosan depended on pH. The swelling degree of PEC-Chi25 was 180 and 420% after three hours of incubation at pH 5.0 and 7.4, respectively (Figure 3B). The PEC-Chi25 hydrogel continued to swell at pH 7.4, while its weight did not change upon further incubation at pH 5.0 (Figure 3B). After three hours of incubation, the PEC-Chi50 hydrogel swelled twice as much at pH 7.4 as it did at pH 5.0 (Figure 3C). The swelling degree of the PEC-Chi75 hydrogel was slightly higher at pH 7.4 than at pH 5.0 (Figure 3D). 

**Figure 4:**
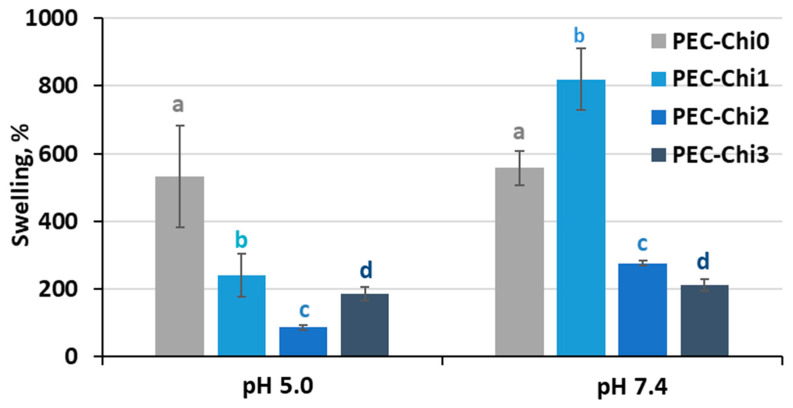
The swelling degree of the PEC-Chi hydrogels after 24 h incubation in Hanks’ solution with pH 5.0 and 7.4 at 37 °C. The values (m ± SD, n = 6) are significantly different between hydrogels at the same pH (*p* < 0.05), as indicated with different letters.

A comparison of hydrogels that reached equilibrium within 24 h of incubation revealed that adding chitosan to the pectin hydrogel decreased its ability to swell in an acidic environment (at pH 5.0) (Figure 4). In a solution with a physiological pH of 7.4, the PEC-Chi25 hydrogel swelled 1.5 times more than the PEC-Chi0 hydrogel, but PEC-Chi50 and PEC-Chi75 hydrogels swelled two times less than PEC-Chi0.

The swelling of pectin gel at pH values higher than 3.5, which is the pKa of D-GalA, occurred because of electrostatic repulsion between pectin chains [25]. It has previously been shown that pectin hydrogels in a phosphate-buffered solution (PBS) swelled more with increasing pH [26,27]. Increasing the pH of PBS causes gel swelling enhancement due to an increase in the number of dissociated COO^−^ groups in pectin, which is followed by electrostatic repulsion of the chains and the exchange of cross-linking calcium ions for sodium ions [25,28]. Calcium ions in Hanks’ solution appeared to produce extra intermolecular cross-links, stabilizing the gel network and keeping it from intensive swelling. It was found that changing the type of gel network as a result of the inclusion of chitosan affected the pH dependence of the swelling of the pectin–chitosan hydrogel. The most pH-sensitive was the PEC-Chi25 hydrogel, which was formed by the calcium ion cross-linking of pectins as well as the cross-linking of pectin and chitosan molecules via a COO^−^ and NH_3_^+^ linkage. Hydrogels that were predominantly formed by polyelectrolyte cross-links (PEC-Chi50 and PEC-Chi75) swelled less at both pH values.

### 2.2. Effect of Chitosan on Protein Adsorption by Pectin–Calcium Hydrogels

**Figure 5:**
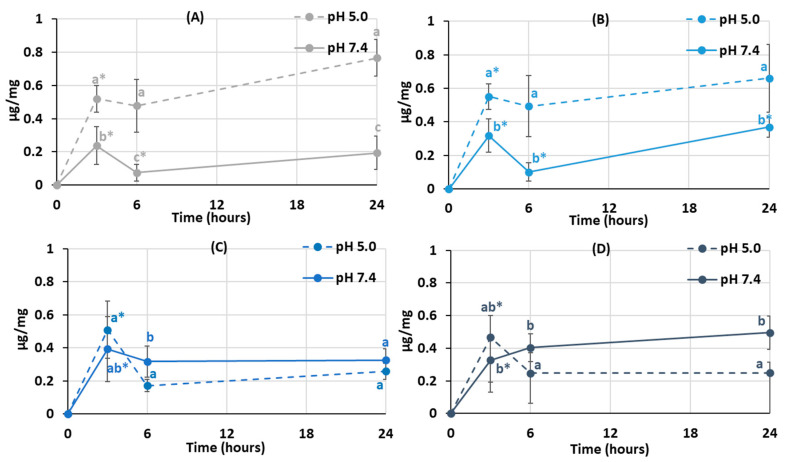
Serum protein adsorption by the PEC-Chi0 (**A**), PEC-Chi25 (**B**), PEC-Chi50 (**C**), and PEC-Chi75 (**D**) hydrogels during incubation in Hanks’ solution supplemented with 10% FBS with pH 5.0 and 7.4 at 37 °C. The values (m ± SD, n = 6) are significantly different between pH conditions (*p* < 0.05), as indicated with different lowercase letters. *—*p* < 0.05 compared to the previous time point.

It was established that protein adsorption by pectin–chitosan hydrogels depended on the pH of the incubation medium. The PEC-Chi0 hydrogel adsorbed 0.5 μg/mg of serum proteins during 3 h of incubation at pH 5.0 in Hanks’ solution supplemented with 10% fetal bovine serum (FBS) (Figure 5A). The adsorption of serum proteins by the PEC-Chi0 hydrogel was about 0.2 μg/mg at pH 7.4. Similarly, the PEC-Chi25 hydrogel adsorbed several times more serum proteins at pH 5.0 than at pH 7.4 (Figure 5B). Maximum adsorption of serum proteins by PEC-Chi50 and PEC-Chi75 hydrogels was observed after 3 h of incubation at pH 7.4 (Figure 5C, D). It was shown that the amount of serum proteins adsorbed by PEC-Chi-50 and PEC-Chi-75 hydrogels decreased with further incubation at pH 5.0 for more than 3 h (Figure 5C,D).

**Figure 6:**
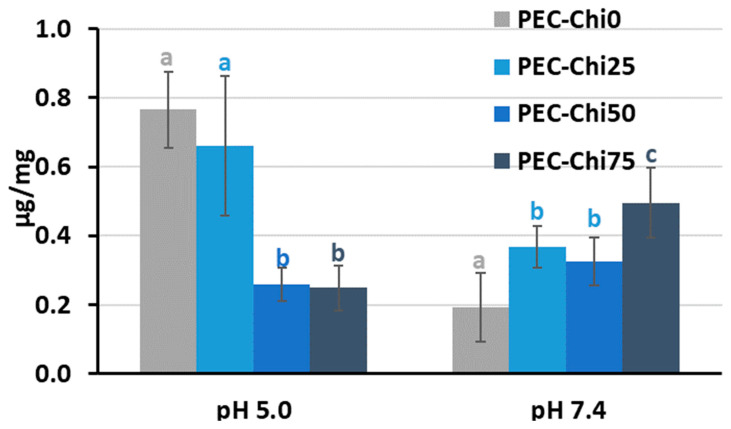
Serum protein adsorption by the PEC-Chi hydrogels after 24 h of incubation in Hanks’ solution supplemented with 10% FBS with pH 5.0 and 7.4 at 37 °C. The values (m ± SD, n = 6) are significantly different between hydrogels at the same pH (*p* < 0.05), as indicated with different letters.

After 24 h of incubation, the addition of chitosan to pectin hydrogel reduced its ability to adsorb serum protein at pH 5.0 while increasing it at pH 7.4 for PEC-Chi75 (Figure 6). At pH 7.4, protein adsorption by PEC-Chi25 and PEC-Chi50 hydrogels was 75–95% higher than that by the PEC-Chi0 hydrogel. A further increase in chitosan content increased the protein adsorption by 2.6 times for PEC-Chi75 when compared to PEC-Chi0.

One of the first things that happens when implanted biomaterial interacts with blood is serum protein adsorption [15]. Hanks’ solution containing FBS was utilized to study serum protein adsorption by pectin–chitosan hydrogels. The protein adsorption’s pH dependency revealed the presence of electrostatic interactions in the adsorption process. Blood serum represents a multi-protein system including albumin, fibrinogen, immunoglobulins, vitronectin, etc., of which serum albumin is the most abundant protein [29]. Because of this, the characteristics of bovine serum albumin (BSA) must be taken into account while attempting to understand how serum proteins adsorb on pectin–chitosan hydrogels. BSA possesses multiple positively charged surface patches in solutions with a pH value lower than BSA’s pI (=4.7) [30]. As was shown in our previous work, the pH of Hanks’ solution shifted from the initial 5.0 to *approx*. 4.0 during the incubation of the pectin gel [12]. Therefore, high protein adsorption under these conditions may be due to the interaction of positively charged groups of protein with negatively charged carboxylic groups of pectin. The number of ionic interactions seemed to decrease as the chitosan and pectin contents increased and decreased, respectively. A net negative charge BSA and pectin macromolecules contribute to electrostatic repulsion and minimal protein adsorption at pH 7.4 since it is known that a BSA solution is negatively charged at pH 7.4 [23]. However, an increase in protein adsorption was detected for the PEC-Chi75 hydrogel, which indicated the presence of a significant amount of NH_3_^+^ groups in PEC-Chi75, which ensured the interaction of chitosan chains with negatively charged sites of BSA. The adsorption of BSA by PEC-Chi75 was calculated to be ca. 200 μg/cm^2^, which exceeded the adsorption of plasma proteins by chitosan-containing materials (5–30 μg/cm^2^) shown earlier in the several studies [17,31,32]. However, protein adsorption by the pectin–chitosan hydrogel (about 2 mg/g) was much lower than that of a chitosan-based material for protein separation (120–165 mg/g) [33]. In general, the data obtained were consistent with studies finding that chitosan coating increased protein adsorption [19].

### 2.3. In Vitro Biocompatibility of Pectin–Chitosan Hydrogels

The activation of the complement system and the induction of blood hemolysis were determined to assess the hemocompatibility of pectin–chitosan hydrogels. Blood samples treated with pectin–chitosan hydrogels at concentrations up to 10 mg/mL did not activate the complement system. Blood samples incubated with hydrogels had a C3a concentration of 5000–8000 ng/mL, similar to those treated with a saline solution (6322 ± 1124 ng/mL). Zymosan, used as a positive control, increased the concentration of C3a to 19,695 ± 2123 ng/mL. The coefficient of induced hemolysis did not exceed 5% when human blood was incubated with pectin–chitosan hydrogels at a concentration of 2–5 mg/mL for 1 h. The coefficient of induced hemolysis when blood is incubated with hydrogels at a concentration of 10 mg/mL is presented in Table 3. As can be seen, hydrogels containing 25 and 50% chitosan induce hemolysis more than pectin hydrogels not containing chitosan.

In general, pectin–chitosan gels demonstrated excellent hemocompatibility, as determined by blood hemolysis and complement activation assays. Excessive hemolysis indicates that blood-contacting materials are incompatible with erythrocytes; thus, biomaterials should induce a hemolysis ratio of less than 5% (Standard Practice for Assessment of Hemolytic Properties of Materials, ASTM F756, 2017 [34]). Many additional studies have already demonstrated strong hemocompatibility for pectin-based biomaterials [35,36,37]. Chitosan was widely recognized to have a hemolytic effect, inducing erythrocyte membrane rupture and hemoglobin release, primarily due to its positively charged amine groups [38]. The findings strongly suggested that the creation of pectin–chitosan cross-links avoided direct contact of the chitosan backbone with the erythrocyte membrane, thereby significantly improving hemocompatibility. The complement system consists of a complex of serum proteins, whose activation aids in the identification of foreign substances and the generation of an innate immune response. Pectin–chitosan hydrogels induced a minor release of C3a when compared to the negative control, independent of chitosan content. These results agree with previously obtained data [39,40].

It was found that the inclusion of chitosan in pectin hydrogel changed the adhesion of peritoneal leukocytes to the gel surface (Table 4). The number of leukocytes that adhered to the PEC-Chi0 hydrogel increased with incubation time. Cells attached to the PEC-Chi0 hydrogel 2.4 and 2.9 times more after 6 and 24 h than after 2 h, respectively. Cell adhesion on the PEC-Chi25 hydrogel was similar to that on the PEC-Chi0 hydrogel. The maximum adhesion of leukocytes after 2 h of incubation, which exceeded the adhesion on PEC-Chi0 by 5.5 times, was observed on the PEC-Chi50 hydrogel. The number of adhered cells on the PEC-Chi50 hydrogel was then reduced until it was comparable to that adhered to the PEC-Chi0 hydrogel after 24 h. The PEC-Chi75 hydrogel attached 4.4 and 3.6 times more leukocytes than the PEC-Chi0 hydrogel after 2 and 6 h, respectively. However, cell adhesion to the PEC-Chi75 hydrogel reduced dramatically, reaching 65% of that of the pectin hydrogel, PEC-Chi0, after 24 h.

**Figure 7:**
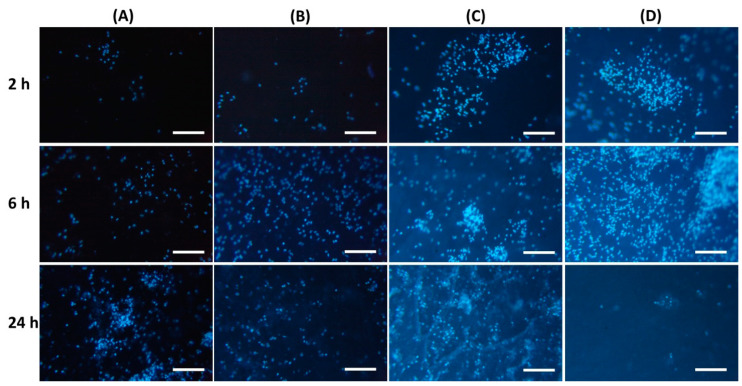
Peritoneal leukocyte adhesion to the surface of PEC-Chi0 (**A**), PEC-Chi25 (**B**), PEC-Chi50 (**C**), and PEC-Chi75 (**D**) hydrogels after 2 (top row), 4 (middle row), and 24 (bottom row) hours of incubation. Cells were stained with DAPI. Magnification 100×. Bar—100 μm.

Representative photographs of the hydrogel surface with adherent cells are shown in Figure 7.

Protein adsorption on the surface of implanted biomaterials affects the intensity of subsequent adhesion and the activation of polymorphonuclear leukocytes and macrophages [15]. According to a number of studies, non-specific protein adsorption promotes cell adhesion [41]. Other authors believe that the so-called “protein coat”, on the contrary, shields the surface of the implanted material, making it less foreign to immune cells [42]. Thus, albumin, the most abundant protein in human blood, has been shown to inhibit the adherence of inflammatory cells such as neutrophils and macrophages, reducing biomaterial-induced inflammatory responses [31,43]. Our findings supported the latter point of view since peritoneal leukocytes adhered the least to the surface of the PEC-Chi75 hydrogel, which had considerable protein adsorption.

### 2.4. Interaction Force of Pectin–Chitosan Hydrogels with Plasma Proteins

**Figure 8:**
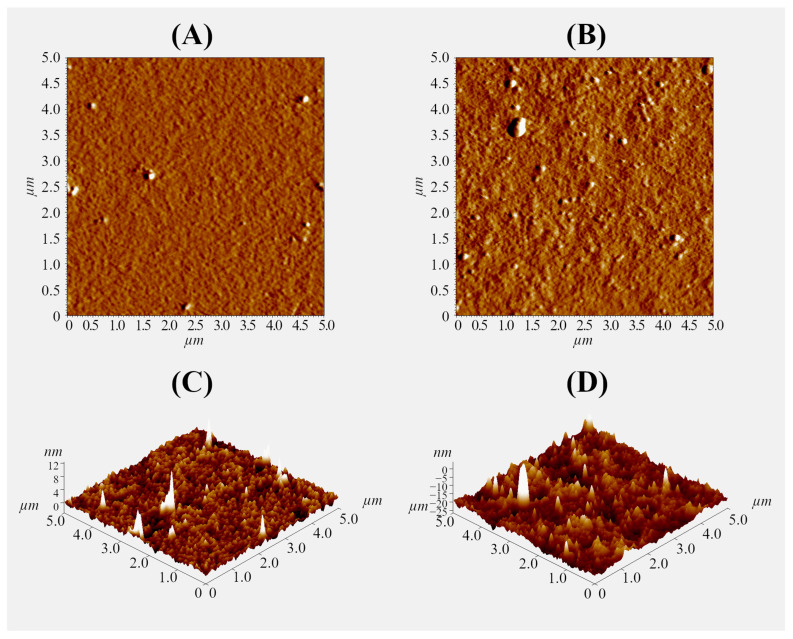
Representative AFM mag images (**A**,**B**) and 3D topographical maps (**C**,**D**) of the surface of PEC-Chi0 (**A**,**C**) and PEC-Chi50 (**B**,**D**) xerogels. The image size is 5 × 5 µm.

A thin gel layer was prepared on the glass surface using 0.1% polysaccharide solutions to further study the interaction of the pectin–chitosan hydrogel with plasma proteins using an optical tweezers approach. The ratio of polysaccharides was varied to obtain a hydrogel layer with a Chi content of 0, 25, 50, and 75% (PEC-Chi0, PEC-Chi25, PEC-Chi50, and PEC-Chi75). The formation of the gel layer was confirmed using atomic force microscopy (AFM). By way of illustration, representative micrographs of the surfaces of PEC-Chi0 and PEC-Chi50 gel-coated glass are shown in Figure 8. The inclusion of Chi made the gel surface rougher. The arithmetic mean profile deviation (Ra) and the height of profile irregularities at ten points (Rz) for PEC-Chi0 were 0.389 ± 0.026 and 1.793 ± 0.138 nm, while for PEC-Chi50, these values were 2.784 ± 0.315 and 10.511 ± 1.785 nm, respectively.

**Figure 9:**
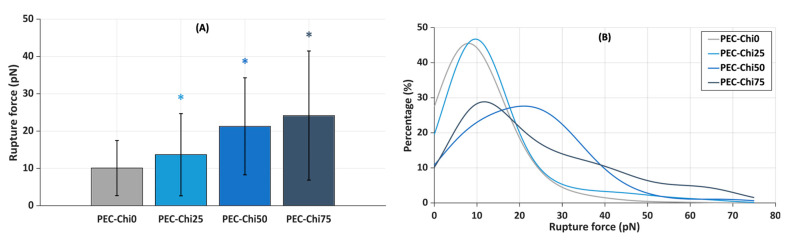
Mean rupture force between PEC-Chi-sensitized glass substrates and microspheres coated with plasma proteins (**A**), and curves approximating the histograms of rupture force distributions (**B**). * *p* < 0.05 vs. PEC-Chi0. The number of measurements for PEC-Chi0, PEC-Chi25, PEC-Chi50, and PEC-Chi75 was 303, 145, 234, and 263, respectively.

Next, the slides coated with the gel layer were incubated in a suspension of plasma protein-coated microspheres that were trapped by a laser beam to determine the force required to lift them off the gel surface. It was found that the inclusion of chitosan increased the force required to detach plasma protein-coated microspheres from the hydrogel surface. The mean rupture force for plasma protein-coated microspheres from the PEC-Chi0 hydrogel was 10.1 ± 7.4 pN (Figure 9A). The rupture of plasma protein-coated microspheres from PEC-Chi25 required 36% more force than that from the PEC-Chi0 hydrogel. A further increase in chitosan content increased the rupture force by 2.1 and 2.4 times for PEC-Chi50 and PEC-Chi75, respectively, when compared to PEC-Chi0.

The curves approximating the rupture force histograms for PEC-Chi0- and PEC-Chi25-covered glass substrates were comparable, with a clear peak in the 10 to 20 pN range in both cases (Figure 9B). The proportion of experiments in which the rupture force exceeded 25 pN increased significantly with increasing chitosan content in the gel layer. Thus, the optical tweezers method’s results were compatible with the findings on serum protein adsorption during the incubation of pectin–chitosan hydrogels in Hanks’ solution supplemented with FBS at pH 7.0.

A variety of methods have been developed to measure non-specific protein adsorption on surfaces. Several approaches that allow for the monitoring of the adsorption process have been utilized in studies devoted to protein behavior at the solid–liquid interface, such as ellipsometry, reflectometry, surface plasmon resonance, total internal reflection fluorescence spectroscopy, γ-photon spectroscopy, quartz crystal microbalance, and atomic force microscopy [22,23]. Despite the importance of these techniques, determining the force of protein–surface interactions in addition to quantifying the amount of adsorbed protein may be crucial for better understanding protein adsorption on biomaterials. In the present study, the optical tweezers method was used for the first time to evaluate the interaction of pectin and pectin–chitosan hydrogels with protein by the example of blood plasma. The process is based on optical trap formation after the laser beam has passed through the optical system and becomes highly focused [44]. Optical trapping is conditioned by the scattering and gradient forces. The first one acts on a particle in the focus along the direction of beam propagation. The gradient force, which is oriented toward the center of the focus and is proportional to the electric field’s gradient, makes it possible to precisely hold and move a dielectric particle in fluid, such as a glass microsphere, bacterial or yeast cell, or polystyrene microsphere. The beam diffracts as it passes through the particle and ultimately strikes the quadrant photodetector. Optical tweezers have previously been used for such molecular pairs as protein antigen–antibody (5–30 pN), carbohydrate antigen–antibody (25 pN), and lipopolysaccharide–monoclonal antibody (40–60 pN), *a*-mannoside–*E. coli* pilus (1.7 pN), fibronectin and *S. aureus* MSCRAMMs (25 pN), etc. [44,45]. The results showed a comparable force of interaction between the pectin–chitosan gel and plasma proteins (10–20 pN). The hydrogel’s binding force to plasma protein-coated particles rose dramatically as chitosan concentration increased. Theoretically, it can be assumed that the inclusion of chitosan in pectin hydrogel could lead to both an increase in the number of protein–surface bonds and a change in the type of bonds to stronger ones.

## 3. Conclusions

In the present study, it was shown that chitosan modifies the mechanical properties of pectin–calcium hydrogel, such as hardness and the cohesiveness-to-adhesiveness ratio. Chitosan in the pectin-calcium hydrogel caused pH-sensitive swelling in Hanks’ solution. A hydrogel consisting of 75% chitosan (PEC-Chi75) adsorbed serum proteins in a physiological environment at pH 7.4 to a greater extent than other hydrogels. PEC-Chi75’s strong adsorption capacity was related to lower peritoneal leukocyte adherence to its surface when compared to other hydrogels, showing improved biocompatibility. Using the optical tweezers approach, it was shown that the force of interaction between pectin–chitosan hydrogels and plasma proteins increased from 10 to 24 pN with increasing chitosan content from 0 to 75%. Thus, the properties of the pectin–calcium hydrogel, which determine interactions with body tissues after implantation, are improved by the addition of chitosan, making the pectin–chitosan hydrogel a promising candidate for smart biomaterial development.

## 4. Materials and Methods

### 4.1. Preparation and Measuring Mechanical Characteristics of Pectin–Chitosan Hydrogels

**Figure 10:**
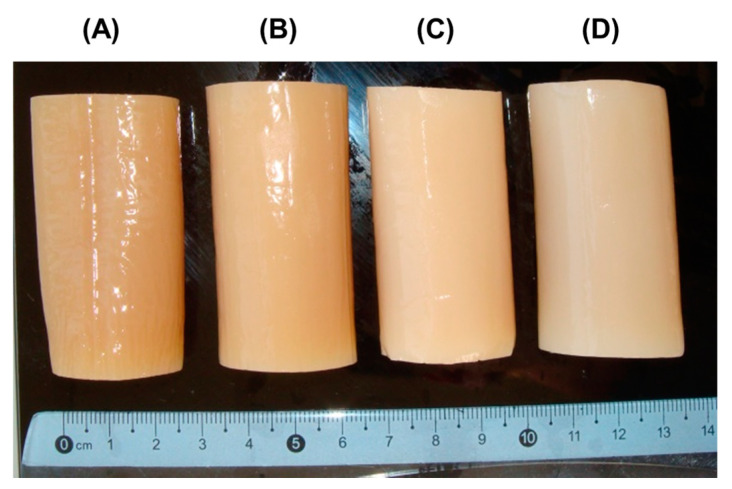
Representative images of PEC-Chi0 (**A**), PEC-Chi25 (**B**), PEC-Chi50 (**C**), and PEC-Chi75 (**D**) hydrogels formed in a dialysis bag.

The pectin–calcium hydrogel (PEC-Chi0) was prepared using external gelation of a 4% solution of low-methyl-esterified apple pectin AU701 (Herbstreith & Fox GmbH, Nuremberg, Germany) with a GalA content of 89.5% and a molecular weight of 401 kDa using the method described previously [12]. Pectin–chitosan hydrogels PEC-Chi25, PEC-Chi50, and PEC-Chi75 were prepared by mixing 4% solutions of apple pectin AU701 and chitosan of a degree of deacetylation of 85% and a molecular weight of 50–310 kDa (Orison Chemical Ltd., Tianjin, China) in a ratio of 4:0, 3:1, 2:2, and 1:3 (volume/volume), respectively. For this, twenty grams of apple pectin were dissolved in 500 mL of distilled water, and twenty grams of chitosan were dissolved in 500 mL of 0.4 M hydrochloric acid. The polysaccharide solutions were mixed in the given amounts and heated to 90 °C with continuous magnetic stirring (200 rpm) for 60 min to improve dissolution before cooling to room temperature. Eighty milliliters of the mixtures obtained were poured into dialysis tubes (pore size: 14 kPa) and immersed in a solution of 1.0 M CaCl_2_ (250 mL) for 48 h at 25 °C to obtain cylinder-shaped hydrogels (Figure 10).

Hydrogel samples (9 × 9 × 9 mm) were compressed twice at room temperature with a cylindrical aluminum probe P/25 using the texture analyzer (Texture Technologies Corp., Stable Micro Systems, Godalming, UK). The pre-test, test, and post-test speeds were 5.0, 2.0, and 2.0 mm/s [46]. Four parameters, such as hardness, cohesiveness, springiness, and adhesiveness, were extracted from a force–time graph and calculated using Texture Exponent 6.1.4.0 software (Stable Micro Systems, UK). 

### 4.2. Swelling Characterization of Pectin–Chitosan Hydrogel

Four mg of dry PEC-Chi hydrogel cubes were incubated in Hanks’ solution (NaCl 140 mM, KCl 5 mM, CaCl_2_ 1 mM, MgSO_4_ 0.4 mM, MgCl_2_ 0.5 mM, Na_2_HPO_4_ 0.3 mM, KH_2_PO_4_ 0.4 mM, D-glucose 6 mM, NaHCO_3_ 4 mM) with pH 5.0 and 7.4 supplemented with 10% FBS (Biolot, Saint-Petersburg, Russia) for 3, 6, and 24 h at 37 °C. Hydrogel samples were weighed using a scale (AG245, Mettler Toledo International, Greifensee, Switzerland) after a predetermined time interval [47]. The swelling degree (SD) was determined as

SD% = ((S_1_ − S_0_)/S_0_) × 100,

where S_0_ and S_1_ are the initial weight and weight after a determined incubation time.

### 4.3. Serum Protein Adsorption by Pectin–Chitosan Hydrogel

Dried gel samples were immersed in Hanks’ solution containing 10% of FBS in wells of 12-well plates at 37 °C, as described earlier [13]. Sample aliquots were collected from wells after 3, 6, and 24 h of incubation and centrifuged at 1000× *g* for 20 min at 4 °C, followed by measuring of the protein concentration in the supernatant using the Micro BCA Protein Assay Kit (Thermo Scientific™, Waltham, MA, USA).

### 4.4. Hemolysis Ratio and Complement Activation

Dried gel samples were immersed in whole blood (0.3 mL) to achieve concentrations of 2, 5, and 10 mg/mL and incubated for 1 h at 37 °C. Then, the blood with gel material was centrifuged at 400 × *g* for 20 min at 4 °C to collect 0.1 mL of the supernatant. The optical density of the supernatants was measured at 540 nm. For controls, 100 µL deionized water (positive) and 50 µL saline (negative) were used [13].

Complement activation evaluation was performed by measuring the C3a levels using the Human C3a ELISA kit (Hycult Biotech, Uden, The Netherlands) [13]. 

### 4.5. Peritoneal Leukocyte Adhesion 

Male BALB/c mice weighing 25–30 g were lavaged in the abdomen with 5 mL of PBS to extract peritoneal leukocytes [48]. After centrifuging the cells in saline for 10 min at 400 × g, they were again suspended in Hanks’ balanced solution, which contained 10% FBS and 25 mM HEPES (pH 7.4) (Sigma-Aldrich, St. Louis, MO, USA). Dried gel samples (9 × 9 × 2 mm, length × width × height) were incubated in 5 mL of cell suspension (2 × 10^6^ cells/mL) at 37 °C for 4 h in a 6-well plate (Greiner Bio-One International GmbH, Kremsmünster, Austria). After incubation, the gel material was treated with 4′,6-diamidino-2-phenylindole (DAPI) (Sigma-Aldrich, St. Louis, MO, USA). The number of adherent leukocytes was counted on three fields of view in each gel sample and normalized by the area of the field of view. The animal study was conducted in accordance with the standards approved by the Ethical Committee of the Komi Science Center of the Russian Academy of Sciences (no. 2022-1003, date of approval: 10 March 2022).

### 4.6. Optical Tweezers Method

#### 4.6.1. Preparing the Glasses

A diamond knife was used to cut rectangular cover slips into equal squares of 24 × 24 mm, which were then immersed in a chrome mixture for one day. The glasses were carefully rinsed the next day in five portions of distilled water before being wiped from the bottom edge with filter paper and dried at room temperature. Dry pectin was dissolved in boiling water, and chitosan plates were dissolved in 0.1 N hydrochloric acid with constant heating until a uniform yellowish gel was obtained. To dilute the concentrates, boiling water or the same acid were used. When flakes fell out, the pectin gels were centrifuged for at least 10 min at 15,000× *g*, after which the supernatants were transferred to separate tubes. To obtain a mixture of polysaccharides, the initial solutions were mixed in a ratio of 1:1, 1:3, or 3:1. The glasses were immersed in boiling solutions of polysaccharides for 5 s, carefully removed, kept in air for 10–20 s, and placed in wide wells of the tablet on circles of filter paper until the moisture completely dried at room temperature. After the gel hardened, the slides were immersed in a 0.3 M CaCl_2_ solution for 30 min to further fix the film, and then removed and washed a second time in three changes of water. After drying the glasses a second time, we glued them to the bottom of the titanium dish using epoxy glue. The workpieces were kept overnight at +37 °C.

#### 4.6.2. Fabrication of Microspheres

A total of 300 µL of citrated human blood plasma was added to 30 µL of a 2.5% suspension of aminated polystyrene microspheres. After keeping the test tube at room temperature for an hour, it was transferred to the refrigerator (+4–6 °C) overnight. In the morning, they were centrifuged for 5 min at 10,000× *g*, the supernatant was removed, the microspheres were washed once with PBS, and the sediment was diluted in 200 μL of fresh PBS with the addition of 0.01% sodium azide. A JFC-1600 vacuum spraying system and a JEOL JSM-6510LV scanning electron microscope (JEOL, Peabody, MA, USA) with an accelerating voltage of 20 kV were used to control the diameter of the microspheres. The microsphere diameter was measured at 2500× magnification using ImajeJ 1.54g software. The average size of the plasma protein layer on microspheres was 1.2 nm. The prepared microspheres were stored in the refrigerator at (+4–6 °C).

#### 4.6.3. Registration of Rupture Forces

To evaluate the interaction forces in the microsphere–substrate model system, JPK NanotrackerTM optical tweezers (JPK, Berlin, Germany) based on an yttrium garnet source with a wavelength of 1064 nm were used. Before the experiment, 2.5 mL of a 0.9% NaCl solution and 2–4 μL of a microsphere suspension were poured into dishes with glasses coated with polysaccharides. The suspension was thoroughly mixed, and the dish was placed on the piezo stage. All work was carried out at +23… +25 °C. Using a laser beam (P = 2.5 W), the microsphere was brought to the bottom of the dish so that the distance between them was 1 μm. The dish was sequentially moved in the direction of the trapped microsphere until the moment of their contact, which was indicated by three successive leaps in the signal chronogram. A second later, the process of retracting the piezo stage in the opposite direction was started in a semi-automatic mode at a speed of about 150 nm/s. The moment the connection was broken was detected by an abrupt change in the signal on the chronogram. To recalculate the primary detector signal into force units, previously obtained calibration coefficients were used (detector sensitivity: 6.4 mV/nm, trap stiffness: 0.26 pN/nm). Histograms were approximated by continuous distribution functions using kernel density estimation [49].

### 4.7. Atomic Force Microscopy

Glasses coated with polysaccharide xerogels prepared as described in Section 4.6 were used to image the surfaces using an A Ntegra Prima atomic force microscope (NT-MDT, Zelenograd, Russia). The samples were scanned in the air in a semi-contact mode using NSG30 cantilevers (NT-MDT, Zelenograd, Russia) at a scan rate of 1 Hz and an image resolution of 512 × 512 pixels. AFM image processing and surface roughness calculations were carried out using Image Analysis of Nova_Px v.3.4.0 software.

### 4.8. Statistical Analysis

The results were expressed as the mean ± standard deviation. This was applied to determine statistically significant differences in hydrogel characterization, swelling, protein adsorption, hemolysis, and complement activation, which were evaluated using a one-way ANOVA with Tukey’s test. An ANOVA for repeated measurements was used to determine statistically significant differences in experiments that involved mouse peritoneal cells. Data obtained using laser tweezers were analyzed using the R program and MatLab 7.0 and Statistica 12 software. Values of *p* ≤ 0.05 were considered statistically significant.

## Figures and Tables

**Table 1:** Mechanical properties of PEC-Chi hydrogels.

Hydrogel	Hardness (N)	Springiness	Cohesiveness	Adhesiveness (mN)
PEC-Chi0	8.9 ± 0.6 ^a^	0.81 ± 0.04 ^a^	0.36 ± 0.14 ^a,b,c^	38.4 ± 10.6 ^a^
PEC-Chi25	16.2 ± 1.1 ^b^	0.70 ± 0.05 ^b^	0.45 ± 0.07 ^a,c^	75.5 ± 9.9 ^b^
PEC-Chi50	6.9 ± 0.3 ^c^	0.86 ± 0.02 ^a^	0.30 ± 0.05 ^b^	41.0 ± 9.1 ^a^
PEC-Chi75	1.9 ± 0.2 ^b^	0.94 ± 0.02 ^c^	0.48 ± 0.05 ^c^	22.1 ± 3.5 ^c^

The values (m ± SD, *n* = 6) are significantly different between hydrogels (*p* < 0.05), as indicated with different lowercase letters.

**Table 2:** Weight (g) of PEC-Chi hydrogels during incubation in distilled water at 4 C for 3 days.

Hydrogel	Initial	After 1 Day	After 2 Days	After 3 Days
PEC-Chi0	0.80 ± 0.02 ^a^	0.78 ± 0.02 ^a^	0.79 ± 0.03 ^a^	0.80 ± 0.02 ^a^
PEC-Chi25	0.80 ± 0.02 ^a^	0.75 ± 0.03 ^b^	0.74 ± 0.03 ^b^	0.73 ± 0.03 ^b^
PEC-Chi50	0.80 ± 0.02 ^a^	0.75 ± 0.02 ^b^	0.75 ± 0.01 ^b^	0.75 ± 0.02 ^b^
PEC-Chi75	0.74 ± 0.02 ^a^	0.69 ± 0.02 ^b^	0.70 ± 0.02 ^c^	0.71 ± 0.02 ^b^

The values (m ± SD, n = 10) are significantly different between different time points (*p* < 0.05), as indicated with different letters.

**Table 3:** The effect of PEC-Chi hydrogels (10 mg/mL) on hemolysis in vitro.

Samples	OD (540 nm)	Hemolysis Ratio (%)
Distilled Water (Positive control)	2.10 ± 0.84	100
0.9% NaCl (Negative control)	0.18 ± 0.02	0
PEC-Chi0	0.23 ± 0.05	2.08 ± 1.37 ^a^
PEC-Chi25	0.28 ± 0.02	5.47 ± 1.72 ^b^
PEC-Chi50	0.31 ± 0.04	6.67 ± 1.21 ^b^
PEC-Chi75	0.27 ± 0.01	4.75 ± 2.5 ^a,b^

OD—optical density. The hemolysis induction coefficient is expressed as a percent. The values (m ± SD, n = 6) are significantly different between hydrogels (*p* < 0.05), as indicated with different lowercase letters.

**Table 4:** A number of peritoneal leukocytes adhered to the surface (mm^2^) of PEC-Chi hydrogels.

Hydrogel	Incubation 2 h	Incubation 6 h	Incubation 24 h
PEC-Chi0	235 ± 107 ^a^	574 ± 177 ^a^*	703 ± 178 ^a^
PEC-Chi25	244 ± 118 ^a^	923 ± 453 ^a^*	547 ± 117 ^a^*
PEC-Chi50	1304 ± 163 ^b^	1158 ± 234 ^a^	733 ± 162 ^a^*
PEC-Chi75	1025 ± 301 ^c^	2041 ± 941 ^b^*	458 ± 223 ^b^*

The values (m ± SD, n = 8) are significantly different between hydrogels (*p* < 0.05), as indicated with different lowercase letters. *—*p* < 0.05 compared to the previous time point.

## Data Availability

The data that support the findings of this study are available from the corresponding author upon reasonable request.
